# Impact on parents of bronchiolitis hospitalization of full-term, preterm and congenital heart disease infants

**DOI:** 10.1186/1471-2431-12-171

**Published:** 2012-10-31

**Authors:** Alexandre Lapillonne, Antoine Regnault, Véronique Gournay, Jean-Bernard Gouyon, Hélène Gilet, Daniela Anghelescu, Tatiana Miloradovich, Benoit Arnould, Guy Moriette

**Affiliations:** 1 Université Paris Descartes, Paris, 75006, France; 2 APHP Necker Hospital, Paris, 75015, France; 3 MAPI Consultancy, Lyon, 69003, France; 4 EA4275 SPHERE Biostatistique, Pharmacoépidémiologie et Mesures Subjectives en Santé, Université de Nantes, Nantes, 44035, France; 5 Service de cardiologie pédiatrique, Centre Hospitalier Universitaire, Nantes, 44093, France; 6 Centre Hospitalier Universitaire de la Réunion, Groupe Hospitalier Sud Réunion, Centre d'Etudes Périnatales de l'Océan Indien, Saint-Pierre, 97448, La Réunion; 7 Medical Department, Abbott France, Rungis, 94528, France; 8 Service de Médecine Néonatale de Port-Royal, Groupe Hospitalier Cochin, AP-HP, Paris, 75014, France; 9 Faculté de Médecine, Université Paris Descartes, Paris, 75006, France

**Keywords:** Bronchiolitis, Family, Infant care, Hospitalization

## Abstract

**Background:**

The objective of this work was to explore the impact on parents of the bronchiolitis hospitalization of their infant using the Impact of Bronchiolitis Hospitalization Questionnaire (IBHQ©).

**Methods:**

Four hundred sixty-three infants aged less than 1 year and hospitalized for bronchiolitis were included in a French observational study during the 2008–2009 season. Parents were asked to complete the IBHQ at hospital discharge and 3 months later. IBHQ scores, ranging from 0 (no impact) to 100 (highest impact), were compared according to gestational age (full-term, 33–36 wGA, ≤ 32 wGA) and the presence of congenital heart disease (CHD). The potential drivers of impact were explored using multivariate linear regressions.

**Results:**

The study included 332 full-terms, 71 infants born at 33–36 wGA, and 60 at ≤ 32 wGA; 28 infants had a CHD. At hospital discharge, 9 of the 12 IBHQ mean scores were above 40, indicating a marked impact on parents. Three months later, all mean scores were lower but 5 were still greater than 40. At discharge, the length of hospitalization had a significant effect on IBHQ worries and distress, fear for future, guilt and impact on daily organization scores (p<0.01); the parents’ educational level had a significant effect on IBHQ worries and distress, fear for future, impact on daily organization and financial impact scores (p<0.05). The only statistically significant difference found between the parents of preterm and full-term infants was for the physical impact score at discharge (p=0.004).

**Conclusions:**

Bronchiolitis hospitalization has conspicuous emotional, physical and organizational consequences on parents and siblings, which persist 3 months after hospital discharge. The main drivers of the impact were length of hospital stay and parents’ educational level, while infants’ gestational age or the presence of a CHD had little influence.

## Background

Bronchiolitis is a viral obstructive bronchial disease occurring in epidemics in infants aged 1–24 months, and manifesting as dyspnoea with tachypnoea, restricted expiration, chest hyperinflation and respiratory distress potentially interfering with feeding; auscultation is dominated by crepitant or subcrepitant rales, rapidly followed by sibilant rales and wheezing [1]. Bronchiolitis is most often caused by respiratory syncytial virus (RSV) [2]. It usually affects infants less than 2 years old, with a peak age of 3–6 months; it is the main cause of hospitalization during the first year of life, therefore exposing many families to the risk of having their infant admitted to hospital [3].

Being hospitalized is difficult for infants but it is also a stressful event for parents [4,5]. The impact on parents of their infant’s hospitalization has been widely studied in the context of pediatric intensive care units (PICU) [6-10]; this research confirmed the distressing impact that this experience can have on parents and siblings. Hence examining the impact on parents of the hospitalization of their infant for a cause as frequent as bronchiolitis is of particular relevance.

Prematurity and congenital heart diseases (CHD) are major risk factors of bronchiolitis hospitalization [3,11-13]. The hospital admission of these infants at-risk may have a specific impact on parents, which is worth investigating. Furthermore, in the context of a hospitalization for bronchiolitis, many factors are likely to affect parents’ experience (e.g. demographics, child’s medical history, or in-ward care). Hence, exploring the drivers of this impact would enable a better understanding of its various levers.

Even though several self-administered questionnaires evaluate the impact of an infant’s illness on the family, none of them specifically measures the distress of parents whose children are hospitalized for bronchiolitis. The Impact of Bronchiolitis Hospitalization Questionnaire (IBHQ^©^) was thus developed to specifically evaluate the impact on parents of the hospitalization of their child for bronchiolitis. The questionnaire was then validated in an observational study in infants aged less than 1 year and hospitalized for bronchiolitis only.

Based on this validation study, the objective of this work was to explore the various facets (emotional, physical, organizational, financial, etc.) of the impact on parents of the hospitalization of their infant for bronchiolitis. We also aimed to specifically evaluate the impact in different populations at-risk (preterm infants and infants with CHD) and to further assess the potential drivers of this impact.

## Methods

### Study design

A multicenter, longitudinal, prospective, observational study was conducted in France from October 2008 to July 2009. Its primary objective was to finalize and validate the IBHQ^©^; exploring the impact on parents of the bronchiolitis hospitalization of their infant was a secondary objective of the study. Eighty-six attending physicians experienced in the management of infants hospitalized for bronchiolitis (49 pediatricians, 21 neonatologists, 3 pediatric cardiologists and 13 pediatric pneumologists) from 77 hospitals (including 13 academic centres) all over the French territory, were involved in this study. Each physician was asked to recruit 7 infants each, with the following distribution: 3 preterm infants (birth at < 37 weeks of gestational age, wGA); 3 full-terms; and 1 infant with CHD. This distribution was meant to ensure that the study sample would include enough infants from these three key populations; however, because of recruitment difficulties, this constraint was released during the course of the study (about half of children were recruited after the removal of distribution recruitment requirement).

Inclusion criteria were: infants less than 1 year of age, hospitalized for bronchiolitis only, and not participating in another clinical study; parents able to read, understand and complete a questionnaire in French. The infants were recruited at the study visit preceding hospital discharge.

Investigators completed a case report form including data on the bronchiolitis hospitalization - severity, duration, in-ward management - and the infant’s medical history - health care received during the neonatal period and risk factors for bronchiolitis (including CHD, chronic lung disease, and family history of allergy). The study population of infants was divided into three gestational age groups to be further analyzed: full-terms (born at ≥ 37 wGA); 33–36 wGA preterm infants, and very preterm infants (born at ≤ 32 wGA).

Parents were asked to fill-in a questionnaire in the week following hospital discharge and three months later, with no specific requirement regarding which parent should complete the questionnaire. The discharge questionnaire was given to parents by physicians at the end of the inclusion visit, and comprised the IBHQ as well as demographics and hospitalization-related socio-economic questions; the 3-months follow-up questionnaire was sent to parents by mail and included the IBHQ, and socio-economic questions referring to the follow-up of the hospitalization.

### The Impact of Bronchiolitis Hospitalization Questionnaire (IBHQ^©^)

The IBHQ is a self-administered questionnaire specifically developed to comprehensively assess the impact on parents of their infant hospitalization for bronchiolitis [14,15]. The development process followed a rigorous methodology including systematic literature review and several steps of interviews with both clinicians and parents whose infants have been recently hospitalized for bronchiolitis. Two versions were developed: one to be completed soon after hospital discharge (DC), and the second to be completed 3 months later to assess the impact during the follow-up (FU) period. These two versions included similar items that assessed the same dimensions but slightly differed in their wording.

Each IBHQ version comprises thirty core items. All these items were to be completed by all the parents, and allowed the calculation of seven core dimension scores: worries and distress, fear for future, guilt, impact on daily organization, physical impact, impact on behavior with hospitalized infant, and financial impact. In addition to the core items, each IBHQ version comprises sixteen optional items. These items, which only addressed specific situations, were to be completed only by the concerned parents, and allowed the calculation of five additional dimension scores: disturbed breastfeeding, physical reaction of the hospitalized infant, impact on feeding of hospitalized infant, impact on behavior with other children, and siblings’ reaction. The IBHQ-DC and IBHQ-FU were previously proved to be reliable and valid.

All dimension scores range from 0 (no impact) to 100 (highest possible impact of the bronchiolitis hospitalization). A score of 0 corresponds to the normal situation; conversely, any score greater than 0 indicates that the hospitalization had an impact on the corresponding dimension. However, no interpretation rule enables a formal definition of a meaningful impact.

### Statistical analyses

1)*General considerations*

The description of socio-demographic and clinical characteristics was performed for all the eligible infants. The analyses of the impact of bronchiolitis hospitalization were performed on all parents of eligible infants who completed both the IBHQ-DC and the IBHQ-FU questionnaires.

All analyses were performed using SAS statistical software version 9.2 (SAS Institute, Cary, NC, USA).

3)*Assessment of the impact of bronchiolitis hospitalization on parents*

IBHQ-DC and IBHQ-FU scores were compared according to the gestational age group (full-terms, 33–36 wGA, and ≤ 32 wGA) and to the presence of CHD risk factor using univariate analysis of variance (ANOVA).

Multivariate linear regression models with stepwise selection were used to identify factors having an independent statistically significant effect on the impact of bronchiolitis hospitalization on parents. The factors whose effect on impact scores were tested pertained to different areas: infant’s characteristics (age at hospitalization, gender, number of siblings), birth conditions (infant from multiple pregnancy, premature birth, associated CHD, Apgar score, hospitalization in neonatology units, supplemental oxygen required for > 24h, mechanical and/or non-invasive ventilation, supplemental oxygen-dependency at 28 days and at 36 wGA), parent’s characteristics (age, gender, education level, professional status), and characteristics of the bronchiolitis hospitalization (duration of hospitalization, department of hospitalization, infusion or parenteral nutrition, enteral nutrition, oxygen therapy, assisted ventilation, non-invasive ventilation, respiratory physiotherapy) as well as of the end of hospitalization and follow-up period (residual respiratory signs at discharge, weight loss, digestive problems, respiratory physiotherapy, oxygen prescription, corticosteroids or bronchodilator prescription). The strength of the link between each variable retained in the multivariate regression after the stepwise selection and the impact of bronchiolitis hospitalization on parents was evaluated using parameter estimates: for continuous variables, the parameter estimate corresponds to the change in the score for a change of 1 unity of the explanatory variable, all other things being equal; for categorical variables, it corresponds to the difference in score for the associate category compared to the reference category, all other things being equal.

### Ethics

The study was conducted in accordance with the principles established in the Declaration of Helsinki and in compliance with local regulatory requirements. The appropriate national authorities and institutional review boards approved the protocol before study commencement. Only parents that returned the signed informed consent form took part in the study.

## Results

### Description of the population

Four hundred and seventy infants hospitalized for bronchiolitis only were included in the study; of these, 7 did not meet one of the inclusion criteria and were not included in the analyses. The parents of eligible infants completed 368 IBHQ-DC and 339 IBHQ-FU; 315 parents completed both IBHQ-DC and IBHQ-FU.

Of the 463 eligible infants, 75% were less than 5 months old and 72% had at least one sibling (Table 1). The study included 28 (6%) CHD infants, of which 2 (7%) born at 33–36 wGA and 3 (11%) at ≤ 32 wGA. Among the 435 infants without CHD, 69 (16%) were born at 33–36 wGA and 57 (13%) at ≤ 32 wGA. One hundred and forty seven infants (32%) had been hospitalized in neonatology units at birth, with a mean duration of stay of 12 days for full-term infants (N=33) and 41 days for preterm infants (N=114) (Table 2). The mean duration of bronchiolitis hospitalization was 6.6 days, with 74% of infants hospitalized in general pediatrics only, 14% in PICUs, and 7% in neonatology units without intensive care facilities (Table 1). Most of the parents who completed the questionnaire were mothers; only 26 fathers (5.6%) completed the discharge questionnaire, 21 discharge questionnaires (4.5%) were completed by both parents, and the information on which parent completed the IBHQ was missing for the other questionnaires. Fathers were slightly older (mean of 33.6 years and 30.1 years for mothers) and more often full-time employed than mothers (65.4% and 33.1% for mothers), while mothers were more often homemakers (42.5%).

**Table 1:** Characteristics and birth conditions of the infants included in the study and characteristics of bronchiolitis hospitalization

		**Eligible infants (N=463)**
Age at hospitalization (months)	Mean (SD)	3.3 (2.7)
	Median (Q1 – Q3)	2.5 (1.3 – 5.0)
	Min – Max	0.3 – 12.0
Sex – n (%)	Male	254 (54.9)
Siblings – n (%)	0	120 (25.9)
	1 or more	335 (72.4)
Multiple pregnancies – n (%)		36 (7.8)
Gestational age – n (%)	Infant with CHD (N=28) – n (%)	
	Full-term	23 (82.1)
	33-36 wGA	2 (7.1)
	≤ 32 wGA	3 (10.7)
	Infant without CHD (N=435) – n (%)	
	Full-term	309 (71.0)
	33-36 wGA	69 (15.9)
	≤ 32 wGA	57 (13.1)
Chronic lung disease – n (%)		31 (6.7)
Family’s allergy history – n (%)		120 (25.9)
Hospitalization in neonatal unit – n (%)		147 (31.8)
Duration of hospitalization (days)	Mean (SD)	6.6 (4.6)
	Median (Q1 – Q3)	5.0 (4.0 – 8.0)
	Min – Max	1.0 – 46.0
Hospitalization department (%)	General pediatrics (only)	73.9
	Intensive care department	14.3
	Neonatal unit (but not intensive care)	7.1
	Other department	4.8
RSV test (%)		91.8
	RSV-positive	72.0
Medications used (N=330)^a^ (%)	Bronchodilators	57.3
	Antibiotics	57.3
	Corticosteroids	31.5
	Anti-reflux treatment	20.0
Non-medicated treatments (N=455) (%)	Respiratory physiotherapy	95.6
	Infusion or parenteral nutrition	37.6
	Oxygen therapy	64.6
	Enteral nutrition	29.9
	Non-invasive ventilation	13.0
	Assisted ventilation	4.8

^a^ Several medicated or non-medicated treatments could be reported.

**Table 2:** Characteristics of the hospitalization in neonatal unit at birth

	**Eligible infants hospitalized in neonatal unit at birth (N=147)**	
	**Full-term (N=33)**	**Preterm (N=114)**
Duration of hospitalization (days)		
Mean (SD)	12.1 (17.4)	40.6 (37.7)
Median (Q1 – Q3)	7.0 (4.0 – 16.0)	28.0 (12.0 – 60.0)
Min – Max	1.0 – 90.0	7.0 – 222.0
Supplemental oxygen >24h (%)	39.4	55.3
Mechanical ventilation (%)	24.2	47.4
Non-invasive ventilation (%)	18.2	55.3
Supplemental oxygen requirement at 28 days (%)	3.0	20.2
Supplemental oxygen requirement at 36 wGA (%)	3.0	13.2

### Impact of bronchiolitis hospitalization on parents at hospital discharge and three months later

IBHQ scores at hospital discharge ranged between 66.6 (worries and distress) and 21.4 (financial impact); nine of the twelve mean scores were above 40 (Table 3). At follow-up, the mean scores ranged between 59.0 (fear for future) and 13.3 (financial impact). All mean scores were lower three months after hospital discharge, with 5 of them being still greater than 40.

**Table 3:** Description of IBHQ scores at hospital discharge and at the three month follow-up

	**IBHQ score**	**Discharge**		**Follow-up**	
		**N**	**Mean (SD)**	**N**	**Mean (SD)**
Core scores	Worries and distress	315	57.5 (23.5)	314	45.4 (26.8)
	Fear for future	306	66.6 (27.2)	308	59.0 (28.3)
	Guilt	313	38.0 (25.5)	314	34.1 (26.6)
	Impact on daily organization	314	62.7 (23.6)	310	18.4 (18.8)
	Physical impact	311	55.4 (21.0)	306	39.6 (24.2)
	Impact on behavior with hospitalized infant	311	55.3 (25.5)	310	45.8 (24.4)
	Financial impact	310	21.4 (19.4)	315	13.3 (14.0)
Optional scores	Disturbed breastfeeding	91	49.5 (33.0)	82	43.5 (35.2)
	Physical reaction of hospitalized infant	298	30.5 (25.9)	302	18.0 (22.0)
	Impact on feeding of hospitalized infant	306	40.1 (26.1)	311	15.6 (18.9)
	Impact on behavior with other children	241	56.6 (27.5)	234	39.8 (28.7)
	Siblings’ reaction	213	63.1 (25.5)	212	57.9 (24.8)

All IBHQ scores range between 0 (no impact) and 100 (highest impact).

### Impact of bronchiolitis hospitalization on parents according to gestational age

**Figure 1:**
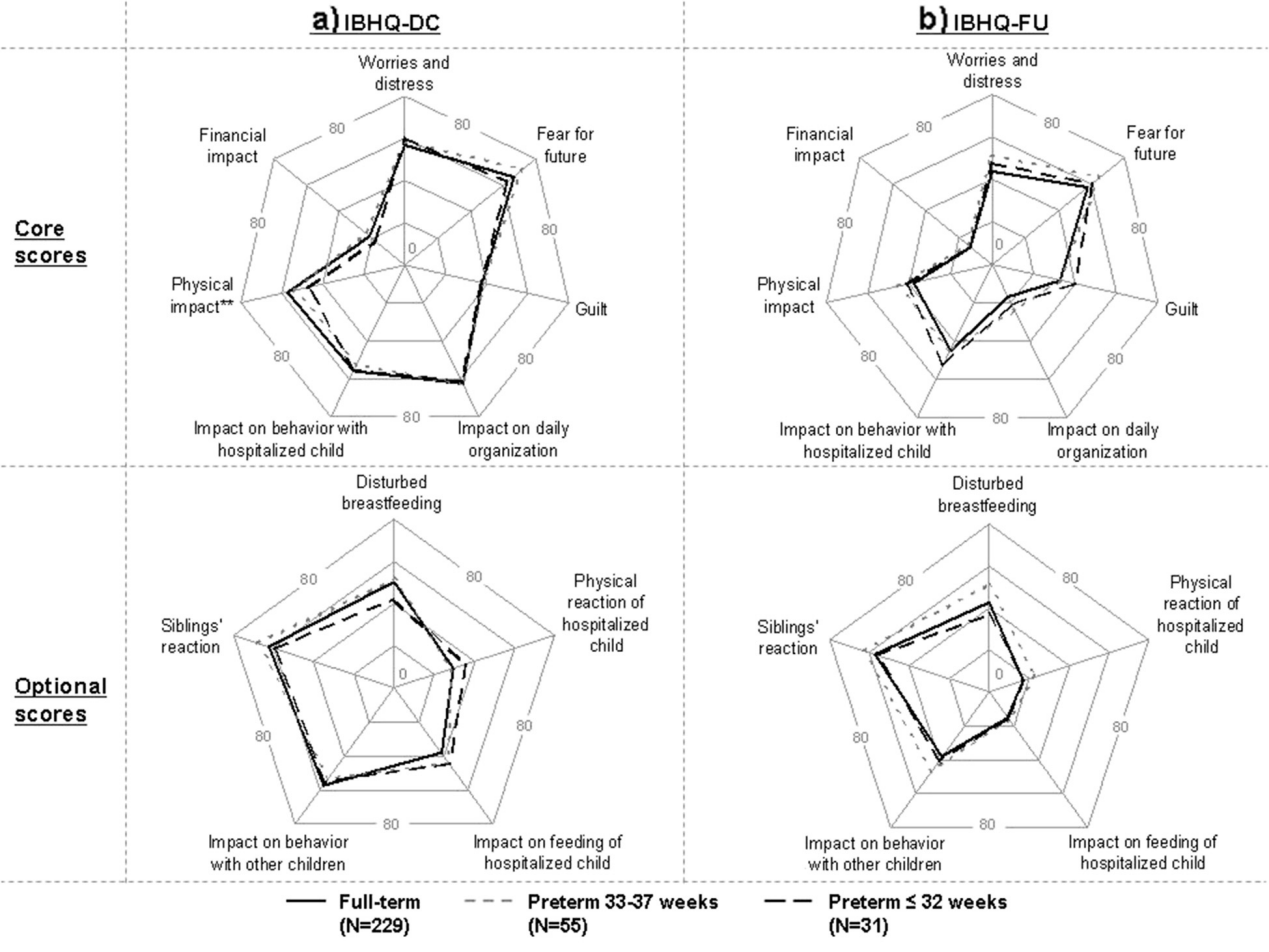
**Comparison of IBHQ scores according to gestational age.** ** P-value from ANOVA < 0.01; Not significant for other scores. Center of the star chart is a mean score of 0 (no impact of bronchiolitis hospitalization), and the extremity of each line is a mean score of 80 (higher impact of bronchiolitis hospitalization).

The IBHQ scores at discharge and follow-up in the three gestational age groups are presented in Figure 1. At discharge, the physical impact score was significantly lower in parents of ≤ 32 wGA infants as compared to parents of more mature infants: mean scores were of 47 for parents of ≤ 32 wGA infants, 54 for parents of 33–36 wGA infants and 57 for full-terms’ parents (p = 0.004); other core scores did not significantly differ between parents of full-term and premature infants. No statistically significant difference was observed in mean optional scores between the three groups.

At follow-up, no statistically significant differences were noticed between the mean scores of parents of full-term, 33–36 wGA and ≤ 32 wGA infants, for either core or optional scores.

### Impact of bronchiolitis hospitalization on parents according to CHD risk factor

**Figure 2:**
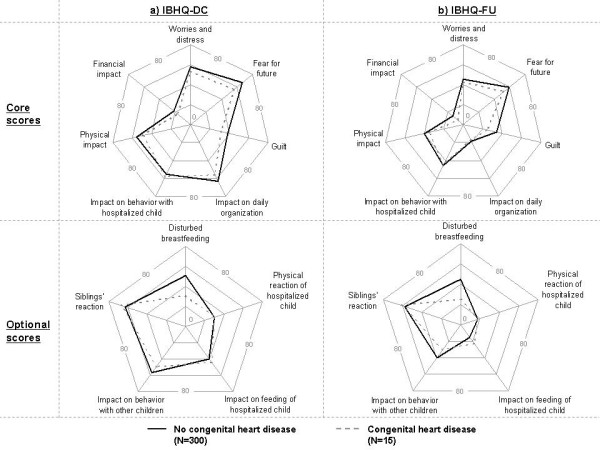
**Comparison of IBHQ scores according to the presence of a CHD.** Not significant for all scores. Center of the star chart is a mean score of 0 (no impact of bronchiolitis hospitalization), and the extremity of each line is a mean score of 80 (higher impact of bronchiolitis hospitalization).

At discharge and follow-up, parents of CHD infants reported lower mean scores, indicating a lower impact as compared to parents of infants without CHD (Figure 2); none of these differences was statistically significant.

### Multivariate model of the impact on parents of bronchiolitis hospitalization

The results of the multivariate models performed on IBHQ scores at discharge are presented in the Tables 4 and 5, for the core and optional scores respectively.

**Table 4:** Multivariate regression models of IBHQ core scores at discharge

		**IBHQ core score - Discharge**						
		**Worries and distress**	**Fear for future**	**Guilt**	**Impact on daily organization**	**Physical impact**	**Impact on behavior with hospitalized infant**	**Financial impact**
		***(R***^***2***^***= 0.137)***	***(R***^***2***^***= 0.122)***	***(R***^***2***^***= 0.094)***	***(R***^***2***^***= 0.122)***	***(R***^***2***^***= 0.056)***	***(R***^***2***^***= 0.014)***	***(R***^***2***^***= 0.182)***
**Intercept**		59.8	77.1	42.6	38.3	48.4	44.4	−1.1
**Level of education of parent (years)**Ref: Other	≤ 5	−24.7	−11.8	−33.3	24.6			
	6-9	−2.9	−5.4	−28.0	−15.9			
	10-12	−5.8	−8.4	−9.0	−4.0			
	13-15	−15.5	−16.2	−19.8	4.2			
	≥ 16	−18.4	−22.3	−22.2	10.3			
*P-value*		*< 0.001*	*0.004*	*< 0.001*	*< 0.001*			
**Duration of hospitalization (days)**		1.5	1.2		0.9			0.9
*P-value*		*< 0.001*	*0.006*		*0.015*			*0.007*
**Infusion or parenteral nutrition during hospitalization**			6.7					
*P-value*			*0.048*					
**Requirement of supplemental oxygen at 28 days**				−12.9				−19.0
*P-value*				*0.041*				*0.001*
**Non-invasive ventilation during hospitalization**					−12.7			
*P-value*					*0.009*			
**Corticosteroids or bronchodilator aerosols planned after hospitalization**					−6.9			
*P-value*					*0.027*			
**Sex of infant (male)**						−5.3		
*P-value*						*0.033*		
**Gestational age **Ref: ≤ 32 wGA	33-37 wGA					8.4		
	Full-term					12.0		
*P-value*						*0.004*		
**Infant from multiple pregnancy**							11.7	
*P-value*							*0.041*	
**Infant age at hospitalization (months)**								0.5
*P-value*								*< 0.001*
**Respondent **Ref: both parents	Father							15.9
	Mother							−9.9
*P-value*								*< 0.001*

The table includes the parameter estimates for the variables retained in the multivariate regressions after the stepwise selection. For continuous variables, the parameter estimate corresponds to the change in the score for a change of 1 unity of the explanatory variable, all other things being equal; for categorical variables, it corresponds to the difference in score for the associate category compared to the reference category, all other things being equal.

**Table 5:** Multivariate regression models of IBHQ optional scores at discharge

		**IBHQ optional score - Discharge**				
		**Disturbed breastfeeding**	**Physical reaction of hospitalized infant**	**Impact on feeding of hospitalized infant**	**Impact on behavior with other children**	**Siblings’ reaction**
		***(R***^***2***^***= 0.269)***	***(R***^***2***^***= 0.078)***	***(R***^***2***^***= 0.111)***	***(R***^***2***^***= 0.126)***	***(R***^***2***^***= 0.090)***
**Intercept**		43.9	7.8	44.0	64.3	53.0
**Duration of hospitalization (days)**				1.2		
*P-value*				*0.007*		
**Hospitalization department**Ref: Other	General pediatrics (only)		19.6			3.2
	Intensive care		32.3			7.6
	Neonatal unit (but not intensive care)		28.0			−13.1
*P-value*			*0.006*			*0.032*
**Infusion or parenteral nutrition during hospitalization**		26.0				
*P-value*		*< 0.001*				
**Enteral nutrition during bronchiolitis hospitalization**				11.5		
*P-value*				*0.002*		
**Oxygen therapy during bronchiolitis hospitalization**			6.9			
*P-value*			*0.037*			
**Residual respiratory signs after hospitalization**				8.8		
*P-value*				*0.008*		
**Corticosteroids or bronchodilator aerosols planned after hospitalization**		−26.8				
*P-value*		*0.004*				
**Infant age at hospitalization (months)**			0.3		−0.4	
*P-value*			*0.019*		*0.009*	
**No siblings**					−38.5	
*P-value*					*< 0.001*	
**Number of siblings**						5.4
*P-value*						*0.003*

The table includes the parameter estimates for the variables retained in the multivariate regressions after the stepwise selection. For continuous variables, the parameter estimate corresponds to the change in the score for a change of 1 unity of the explanatory variable, all other things being equal; for categorical variables, it corresponds to the difference in score for the associate category compared to the reference category, all other things being equal.

None of the studied factors systematically had a significant impact on all IBHQ scores. Two factors had a statistically significant effect on at least 4 scores: the length of hospital stay and the parents’ educational level. Longer hospitalization was associated with higher scores for worries and distress, fear for future, impact on daily organization, financial impact, and impact on feeding of hospitalized infant. Parents’ educational level had a significant effect on worries and distress, fear for future, guilt, and impact on daily organization scores, with a non linear pattern of association: parents in extreme categories (< 5 and >16 years of education) seemed to experience less emotional impact and higher daily organization impact than parents having 6–15 years of education. Moreover, each IBHQ score was specifically affected by other factors.

As for simple comparisons, the gestational age group was only significantly associated with the physical impact IBHQ scores in the multivariate models.

Results of the multivariate regression analyses at follow-up showed a similar pattern (Additional file 1: Table S1 and Additional file 2: Table S2): only the length of hospitalization duration and the parents’ educational level had a statistically significant impact on at least 4 scores; different other factors specifically affected various IBHQ-FU scores.

## Discussion

This study showed that the hospitalization of an infant for bronchiolitis has a multidimensional impact on its parents as well as on the whole family: emotional and physical impact, impact on daily life organization and parenting role, and impact on siblings. Among the various studied aspects, only the financial impact and to a lesser extent parent’s perception on the physical reaction of the hospitalized infant revealed lower levels of impact. Unsurprisingly, the impact at hospital discharge was consistently stronger than the impact 3 months later; however, the impact 3 months after hospital discharge remained notable for many aspects (especially for emotional consequences, parenting roles, and reactions of siblings).

One of the main challenges of this study was to interpret the IBHQ results, as this is the first study using the IBHQ, and no control group can be defined to compare the specific impact of hospitalization for bronchiolitis with the impact of hospitalization for other reasons since the IBHQ is a specific questionnaire that is not suitable for other populations / pathologies than parents of children hospitalized for bronchiolitis. Given the absence of clear rules for the interpretation of scores, it had to rely largely on the content of the questions. For example, to obtain a value of 40 for the mean guilt score at hospital discharge, parents would have answered “very much” or “extremely” to at least one of the three questions of the dimension (i.e. feeling guilty for leaving the infant in hospital, feeling guilty because of the infant’s bronchiolitis, and feeling lonely). In light of this kind of considerations, it clearly appeared that the observed mean scores, which ranged between 40 and 60 for almost all scores, are indicative of a non-trivial impact.

The impact on parents of the hospitalization of their child was often investigated in the context of severe diseases, in a PICU context [6-10,16]. A literature review revealed the great emotional and physical impact on the parents of hospitalized critically ill children [17]; moreover, these hospitalizations were shown to have important consequences on the whole family. To our knowledge, a single previous study specifically explored the impact on parents of their infant’s hospitalization for bronchiolitis in a small sample (N = 46) of infants hospitalized with RSV-bronchiolitis [18]; this work showed a clear impact on emotional aspects and parenting role that persisted several weeks after hospital discharge. The present study confirmed these key findings in a much larger sample, since most of the results were based on more than 300 infants. Moreover, our results provide a broader spectrum of this impact, by adding physical and daily life organization consequences to the picture as well as by outlining the distressful impact on the siblings of the hospitalized infant.

We also analyzed the impact in different subgroups of parents of infants at high-risk of hospitalization for bronchiolitis, i.e. preterm and CHD infants. The few differences found depending on gestational age were small and did not appear meaningful. As for parents of infants with CHD, unexpectedly, they seemed overall less affected by the bronchiolitis hospitalization. This could indicate a different perception of the bronchiolitis hospitalization in these parents whose infants had already experienced more serious hospitalizations; their reaction to the bronchiolitis hospitalization may be mitigated by their previous difficult experiences, and they may have already created strategies to cope with the burden of their infant’s hospitalization. Nevertheless, these findings should be interpreted with caution given the small observed impact differences as well as the limited number of CHD infants included in the analyses.

We also evaluated the main drivers of the impact of bronchiolitis hospitalizations on parents: none of the factors considered in these analyses consistently affected all the dimensions of the impact. Nonetheless two factors were associated to a higher impact on several aspects: duration of hospitalization and parents’ educational level. Hospital length of stay can be considered as a surrogate marker of the severity of the hospitalization, which has already been shown as a key driver of impact on parents of the hospitalization of their infant in previous studies [4,5,7]. Parents’ level of education equally had a significant effect on many aspects of the impact of hospitalization: worries and distress, fear for future, guilt, and impact on daily organization. However, no clear, consistent pattern was observed; the impact of bronchiolitis hospitalization is obviously dependent on the parents’ level of education, but this relationship is not straightforward. Further research specifically focusing on this issue is warranted in order to get a better understanding of how hospitalizations for bronchiolitis affect parents with different educational levels.

Despite, the population of the present study seemed to be similar to the population of infants generally hospitalized for bronchiolitis, and the in-ward management was consistent with the current medical practice, the representativeness of the study sample could not be formally supported, in the absence of French reference data. The stratified enrolment procedure, which initially imposed the inclusion of preterm and CHD infants, increased the percentage of these cases in our study, and therefore our sample may be slightly different from the general population of infants hospitalized for bronchiolitis. The longer hospital stays observed in this study as compared to those retrieved from the 2005–2006 French National Hospitalizations Database (*Programme de Médicalisation des Systèmes d’Information; PMSI*) could be explained by these differences: the mean length of hospitalization in our study was of 6.6 days while the mean length of hospital stay for bronchiolitis in infants younger than one year was of 4.7 days in the PMSI database.

Some other specific features should also be taken into account when interpreting the results of the present study. The parent who completed the questionnaire was in most of the cases the mother (92%). The parent’s gender doubtlessly generates certain differences in the impact of the hospitalization [5] but the paucity of data collected from fathers in this study does not allow reliable conclusions on the impact on fathers of their infant’s bronchiolitis hospitalization. Also, parents with low educational level might be underrepresented in this study sample because an inclusion criterion required the responding parent to be able to complete the questionnaire alone; this aspect could be particularly important since low educational level of the mother has been shown to be a risk factor for infant’s hospitalization for bronchiolitis [19]. In addition, among the children whose parents accepted to participate in the study, those whose parents eventually completed the discharge questionnaire had less often CHD, had shorter hospitalizations, and less often admitted in intensive care units than children whose parents did not complete the discharge questionnaire, suggesting that they may have presented a less severe level of bronchiolitis. However, even for those parents, the bronchiolitis hospitalization of their children showed a clear impact, which demonstrates that, regardless of the severity of the disease, the hospitalization of the child is not of no consequence for the parents. Also, we could not take into account the potential impact on the follow-up assessment of additional family events that could have occurred in the three month period since discharge. Finally, this study was conducted in France and, even if most of the results are probably applicable to other Western European countries, certain cultural peculiarities might not be replicated in other cultures. In particular, the low financial impact of bronchiolitis hospitalization may be somewhat specific to France, where the costs of hospitalization are fully reimbursed by the social security system.

## Conclusions

The hospitalization of an infant for bronchiolitis was confirmed to seriously impact its parents and family. This impact was shown to be multifaceted and persistent even three months after hospital discharge for several aspects, in particular the emotional dimensions of parents’ impact, the parenting role, and the impact on siblings. The main drivers of this impact were length of hospital stay and parents’ educational level; conversely, infants’ gestational age and presence of a CHD had little influence on the impact on parents of their infant hospitalization for bronchiolitis.

## Abbreviations

CHD: congenital heart disease; IBHQ: Impact of Bronchiolitis Hospitalization Questionnaire; PICU: Pediatric Intensive Care Unit; RSV: Respiratory Syncytial Virus.

## Competing interests

This study was sponsored by Abbott France. AL, JBG, VG and GM received honorarium for their scientific advice and expertise. AR, HG and BA are employees of Mapi Consultancy, consulting company commissioned by Abbott for this study. DA and TM are employees of Abbott.

## Authors' contributions

AL, VG, JBG and GM provided clinical and scientific expertise on bronchiolitis’s disease along the project, in particular for definition of objectives, patient inclusion and non-inclusion criteria validation, patient recruitment, interpretation of results, choice of concepts to be measured, and finalisation of the questionnaire, and critically reviewed the manuscript. AR participated in the validation study design, designed statistical analyses, participated in the interpretation of results, and drafted the manuscript. HG performed statistical analyses, participated in the interpretation of results and helped draft the manuscript. DA and TM participated in the interpretation of data and critically reviewed the manuscript. BA provided scientific expertise on the methodology used to develop the questionnaire and on the design of the study and of the statistical analysis, participated in the item generation of the questionnaire and in the interpretation of the study results, and critically reviewed the manuscript. All authors read and approved the final manuscript.

## Pre-publication history

The pre-publication history for this paper can be accessed here:

http://www.biomedcentral.com/1471-2431/12/171/prepub

## Supplementary Material

Additional file 1 **Table S1.** Multivariate regression models of IBHQ core scores at follow-up. Click here for file

Additional file 2 **Table S2.** Multivariate regression models of IBHQ optional scores at follow-up. Click here for file
