# Supplementary material for: Impact on parents of bronchiolitis hospitalization of full-term, preterm and congenital heart disease infants
Source: BMC Pediatr. 2012 Oct 31;12:171. doi: 10.1186/1471-2431-12-171 (PMC3506487; doi:10.1186/1471-2431-12-171)
Supplement: Additional file 1 — Table S1. Multivariate regression models of IBHQ core scores at follow-up. [file 1471-2431-12-171-S1.doc]

**Table 1**: Multivariate regression models of IBHQ core scores at follow-up

|  |  | **IBHQ core score - Follow-up** | | | | | | | | | | | | | | | | | | |
| --- | --- | --- | --- | --- | --- | --- | --- | --- | --- | --- | --- | --- | --- | --- | --- | --- | --- | --- | --- | --- |
|  |  | **Worries and distress** | | | **Fear for future** | | **Guilt** | | | **Impact on daily organization** | | | **Physical impact** | | | **Impact on behavior with hospitalized infant** | | **Financial impact** | | |
|  |  | ***(R² = 0.151)*** | | | ***(R² = 0.081)*** | | ***(R² = 0.024)*** | | | ***(R² = 0.122)*** | | | ***(R² = 0.017)*** | | | ***(R² = 0.131)*** | | ***(R² = 0.143)*** | | |
| **Intercept** | | 58.8 | |  | 64.4 |  | 38.7 |  | | 21.0 |  | | 35.2 | |  | 57.3 |  | 34.2 | |  |
| **Level of education of parent (years)**  Ref: Other | ≤ 5 | -24.4 | |  | -23.9 |  |  | | | -21.6 |  | |  | | | -31.2 |  | -16.7 |  | |
|  | 6-9 | 1.2 | |  | -6.8 |  |  |  |  | 3.3 |  | |  |  |  | -12.4 |  | -12.8 |  | |
|  | 10-12 | 1.7 | |  | -3.0 |  |  |  |  | -0.9 |  | |  |  |  | 1.2 |  | -8.2 |  | |
|  | 13-15 | -12.8 | |  | -14.0 |  |  |  |  | -7.4 |  | |  |  |  | -4.0 |  | -12.1 |  | |
|  | ≥ 16 | -17.4 | |  | -20.9 |  |  |  |  | -6.4 |  | |  |  |  | -12.6 |  | -9.6 |  | |
| *P-value* | | *< 0.001* | | | *0.004* | |  |  |  | *0.034* | | |  |  |  | *0.021* | | *0.001* | | |
| **Duration of hospitalization (days)**  Ref: ≥ 8 | < 4 | -10.5 | |  |  | |  | | |  | | |  | | |  | |  | | |
|  | 4-5 | -9.1 | |  |  |  |  |  |  |  |  |  |  |  |  |  |  |  |  |  |
|  | 5-7 | -11.4 | |  |  |  |  |  |  |  |  |  |  |  |  |  |  |  |  |  |
| *P-value* | | *0.024* | | |  |  |  |  |  |  |  |  |  |  |  |  |  |  |  |  |
| **Duration of hospitalization (days)** | |  | |  | 1.0 |  |  | | | 1.0 |  | | 0.8 |  | |  | |  | | |
| *P-value* | |  | | | *0.019* | |  |  |  | *< 0.001* | | | *0.025* | | |  |  |  |  |  |
| **Non-invasive ventilation during hospitalization** | |  | | |  | |  | | |  | | |  | | | 11.9 |  |  | | |
| *P-value* | |  |  |  |  |  |  |  |  |  |  |  |  |  |  | *0.016* | |  |  |  |
| **Residual respiratory signs after hospitalization** | |  | | |  | |  | | | 5.6 |  | |  | | | 8.2 |  |  | | |
| *P-value* | |  |  |  |  |  |  |  |  | *0.018* | | |  |  |  | *0.009* | |  |  |  |
| **Digestive problems after hospitalization** | |  | | |  | |  | | |  | | |  | | |  | | 5.6 |  | |
| *P-value* | |  |  |  |  |  |  |  |  |  |  |  |  |  |  |  |  | *0.047* | | |
| **Respiratory physiotherapy planned after hospitalization** | | -8.6 |  | |  | |  | | |  | | |  | | |  | |  | | |
| *P-value* | | *0.014* | | |  |  |  |  |  |  |  |  |  |  |  |  |  |  |  |  |
| **Sex of infant (male)** | |  | | |  | |  | | |  | | |  | | |  | | 4.0 |  | |
| *P-value* | |  |  |  |  |  |  |  |  |  |  |  |  |  |  |  |  | *0.013* | | |
| **Infant age at hospitalization (months)** | |  | | |  | | -0.4 | |  |  | | |  | | |  | |  | |  |
| *P-value* | |  |  |  |  |  | *0.008* | | |  |  |  |  |  |  |  |  |  | | |
| **No siblings** | |  | | |  | |  | | | -6.3 | |  |  | | |  | | -4.1 |  | |
| *P-value* | |  |  |  |  |  |  |  |  | *0.013* | | |  |  |  |  |  | *0.024* | | |
| **Number of siblings** | |  | | |  | |  | | |  | | |  | | | 4.9 |  |  | | |
| *P-value* | |  |  |  |  |  |  |  |  |  |  |  |  |  |  | *< 0.001* | |  |  |  |
| **Respondent**  Ref: both parents | Father |  | | |  | |  | | |  | | |  | | |  | | 0.5 | |  |
|  | Mother |  |  |  |  |  |  |  |  |  |  |  |  |  |  |  |  | -7.8 | |  |
| *P-value* | |  |  |  |  |  |  |  |  |  |  |  |  |  |  |  |  | *0.007* | | |
