# Supplementary material for: Impact on parents of bronchiolitis hospitalization of full-term, preterm and congenital heart disease infants
Source: BMC Pediatr. 2012 Oct 31;12:171. doi: 10.1186/1471-2431-12-171 (PMC3506487; doi:10.1186/1471-2431-12-171)
Supplement: Additional file 2 — Table S2. Multivariate regression models of IBHQ optional scores at follow-up. [file 1471-2431-12-171-S2.doc]

**Table 2**: Multivariate regression models of IBHQ optional scores at follow-up

|  | |  | **IBHQ optional score- Follow-up** | | | | | | | | | | |
| --- | --- | --- | --- | --- | --- | --- | --- | --- | --- | --- | --- | --- | --- |
|  | |  | **Disturbed breastfeeding** | | **Physical reaction of hospitalized infant** | | | | **Impact on feeding of hospitalized infant** | | **Impact on behavior with other children** | **Siblings’ reaction** | |
|  | |  | ***(R² = 0.170)*** | | ***(R² = 0.157)*** | | | | ***(R² = 0.091)*** | |  | ***(R² = 0.022)*** | |
| **Intercept** | | | 81.4 |  | | 76.1 |  | | 30.5 |  |  | 51.4 |  |
| **Level of education of parent (years)**  Ref: Other | | ≤ 5 |  | | 11.5 | |  | | -17.7 |  |  |  | |
|  |  | 6-9 |  |  | 1.8 | |  | | -8.5 |  |  |  |  |
|  |  | 10-12 |  |  | -3.2 | |  | | -10.4 |  |  |  |  |
|  |  | 13-15 |  |  | -9.2 | |  | | -15.5 |  |  |  |  |
|  |  | ≥ 16 |  |  | -1.5 | |  | | -13.6 |  |  |  |  |
| *P-value* | | |  |  | *0.049* | | | | *0.004* | |  |  |  |
| **Assisted ventilation during hospitalization** | | | 29.6 |  | 21.4 | | |  |  | |  |  | |
| *P-value* | | | *0.013* | | *< 0.001* | | | |  |  |  |  |  |
| **Oxygen prescription planned after hospitalization** | | |  | | 48.1 | | |  | 5.6 |  |  |  | |
| *P-value* | | |  |  | *0.019* | | | | *0.023* | |  |  |  |
| **Infant age at hospitalization (months)** | | | -1.3 |  |  | | | |  | |  |  | |
| *P-value* | | | *0.009* | |  |  |  |  |  |  |  |  |  |
| **Number of siblings** | | |  | |  | | | |  | |  | 3.8 |  |
| *P-value* | | |  |  |  |  |  |  |  |  |  | *0.038* | |
| **Respondent**  Ref: both parents | Father | |  | | 29.9 | | |  | 12.0 |  |  |  | |
|  | Mother | |  |  | 15.1 | | |  | 1.1 |  |  |  |  |
| *P-value* | | |  |  | *< 0.001* | | | | *0.048* | |  |  |  |
